# Electrophysiological and behavioural response of *Philaenus spumarius* to essential oils and aromatic plants

**DOI:** 10.1038/s41598-020-59835-1

**Published:** 2020-02-20

**Authors:** Sonia Ganassi, Pasquale Cascone, Carmela Di Domenico, Marco Pistillo, Giorgio Formisano, Massimo Giorgini, Pasqualina Grazioso, Giacinto S. Germinara, Antonio De Cristofaro, Emilio Guerrieri

**Affiliations:** 1 University of Molise, Department of Agricultural, Environmental and Food Sciences, Campobasso, 86100 Italy; 2 National Research Council of Italy, Institute for Sustainable Plant Protection, Department of Biology, Agriculture and Food Sciences, Portici, Na 80055 Italy; 3 University of Foggia, Department of the Sciences of Agriculture, Food and Environment, Foggia, 71122 Italy; 4 University of Modena and Reggio Emilia, Department of Life Sciences, Modena, 41125 Italy

**Keywords:** Environmental sciences, Environmental chemistry, Environmental monitoring

## Abstract

The meadow spittlebug, *Philaenus spumarius*, is a highly polyphagous widespread species, playing a major role in the transmission of the bacterium *Xylella fastidiosa* subspecies *pauca*, the agent of the “Olive Quick Decline Syndrome”. Essential oils (EOs) are an important source of bio-active volatile compounds that could interfere with basic metabolic, biochemical, physiological, and behavioural functions of insects. Here, we report the electrophysiological and behavioural responses of adult *P*. *spumarius* towards some EOs and related plants. Electroantennographic tests demonstrated that the peripheral olfactory system of *P*. *spumarius* females and males perceives volatile organic compounds present in the EOs of *Pelargonium graveolens*, *Cymbopogon nardus* and *Lavandula officinalis* in a dose-dependent manner. In behavioral bioassays, evaluating the adult responses towards EOs and related plants, both at close (Y-tube) and long range (wind tunnel), males and females responded differently to the same odorant. Using EOs, a clear attraction was noted only for males towards lavender EO. Conversely, plants elicited responses that varied upon the plant species, testing device and adult sex. Both lavender and geranium repelled females at any distance range. On the contrary, males were attracted by geranium and repelled by citronella. Finally, at close distance, lavender and citronella were repellent for females and males, respectively. Our results contribute to the development of innovative tools and approaches, alternative to the use of synthetic pesticides, for the sustainable control of *P*. *spumarius* aiming to contrasting the expansion of *X*. *fastidiosa*.

## Introduction

The meadow spittlebug *Philaenus spumarius* L. (Hemiptera: Aphrophoridae) is an extremely common species distributed in the Palaearctics, Nearctics, and in the temperate regions of earth and oceanic islands^1–3^. This species occurs in a variety of habitats such as meadows, abandoned fields, waste ground, roadsides, streamsides, and cultivated fields^2^. Due to its high polyphagy at any stage of development, hundreds of plants have been recorded as hosts in Europe, although a preference for dicots over monocots ones is reported^4,5^. Among dicots, herbaceous Fabaceae, able to fix nitrogen and characterized by a high aminoacid concentration in the xylem sap (*e*.*g*. *Medicago sativa*, *Trifolium* spp., *Vicia* spp.) are favoured hosts^6^. Because of the warm, and dry conditions of Mediterranean areas, where the ground cover vegetation almost completely disappears during summer, adults move from herbaceous plants to woody ones^7^. Nymphs and adults are xylem-sap feeders, able to attack aboveground organs with a preference for actively growing parts^8,9^. Direct damages linked to sap ingestion include a general weakening of the plant, deformation, delayed plant maturity and reduced forage yield^10^. By far more serious is the damage linked to the ability of *P*. *spumarius* to act as a vector of phytopathogens such as *Xylella fastidiosa* subsp. *fastidiosa* agent of the Pierce’s disease of grapevines^11^ and most recently the bacterium *Xylella fastidiosa* strain subsp. *pauca*, infecting olives trees in the Salento Peninsula, Italy^12,13^. This bacterium is the main etiological agent of the “Rapid Decline of the Olive Tree Complex [Complesso del Disseccamento Rapido dell’Olivo (CoDiRO)]” and can be transmitted from infected plants to healthy ones^14^. *Xylella fastidiosa* is persistent in its vectors, including *P*. *spumarius*, making them able to transmit it over a long period^12^. The quick spreading of this mortal disease of olive trees in the South of Italy constitutes a serious threat for the entire Mediterranean Basin.

Within the framework of IPM, a number of studies started on either plant resistance and on sustainable methods for controlling the main vector, *P*. *spumarius*, alternative to the use of synthetic pesticides^13,15^.

Innovative approaches to pest control include the manipulation of the foraging behaviour of the target pest and relative natural enemies. Examples of these innovative approaches are the push-and-pull^16^ and attract-and-kill^17^ techniques that are increasingly applied in modern agriculture. Chemical cues play a crucial role in the host-selection process by herbivorous insects that use them to differentiate between host and non-host plant. The above mentioned control methods are based on the exploitation of specific volatile organic compounds that can be released by specific companion plants or applied to the crop artificially through dispensers^18^. These volatile compounds either concentrate the population of the pest in a specific area of the field where the control could be more effective or repel the pest from the main crop. Moreover, they can lure the natural enemies of target pest enhancing the biological control^19^. The semiochemistry underpinning the role of the companion plants is investigated by chemical analyses, and electrophysiological recordings and behavioural bioassays with extracts of volatile compounds they release.

Essential oils (EOs) are an important source of bio-active volatile compounds^16^ biosynthesized in different plant organs that are able to interfere with basic metabolic, biochemical, physiological, and behavioural functions of insects^20^. EOs have been shown to play a role in direct and indirect plant defences against herbivores and pathogens^21^ and can be rightly considered as a valid sustainable alternative to synthetic insecticides^22–25^. Their rapid degradation in the environment reduces the risk of negative effect on non-target organisms. Moreover, their novel and multiple mode of action reduce the probability of developing resistance^22,26,27^.

Regardless the positive features of these compounds, the knowledge of their effect on important agricultural pests and availability of commercial formulations for immediate use in agriculture is still in its infancy^28^. Conversely, the use of companion plants to enhance the resistance of the main crop to pests is widespreading rapidly all around the world^29^.

Among plants, commonly used to extract EOs, some Labiatae have been reported as occasionally attractive to *P*. *spumarius*^30^, whereas Poaceae and Geraniaceae have been found to be negatively selected by the spittlebug^31^.

Previous studies, demonstrated the presence of basiconic and coeloconic sensilla with a putative olfactory function on the male and female antennae of *P*. *spumarius* adults^32^ and electroantennographic tests demonstrated the capability of adult meadow spittlebug to perceive a wide range of volatile organic compounds (VOCs) including aliphatic aldehydes, alcohols, esters, ketones, terpenoids, and aromatic compounds^33^. In order to enhance the availability of practical tools for the management of the spittlebug populations, in the present study the sensitivity of male and female antennae to EOs of *Pelargonium graveolens* (Geraniaceae), *Cymbopogon nardus* (Poaceae) and *Lavandula officinalis* (Labiatae) was investigated by electroantennography and the behavioural responses of adult insects to EOs and related plants was determined using Y-tube olfactometer bioassays. Moreover the behavioural responses of insects to volatiles emitted by plant was further investigated in wind tunnel bioassays.

## Results

### Electroantennographic recording (EAG)

The mean EAG amplitudes evoked by each dose of the three EOs in males and females collected in the Campobasso area, were not significantly different (p > 0.05) from those of males and females from the Benevento area, respectively (*P*. *graveolens* females: *t* = 0.072–0.551; d.f. 13; P > 0.05; males: *t* = 0.185–0.994; d.f. 12; P > 0.05; *C*. *nardus* females: *t* = 0.462–1.912; d.f. 13; P > 0.05; males: *t* = 0.019–1.769; d.f. 12; P > 0.05; *L*. *officinalis* females: *t* = 0.032–1.161; d.f. 12; P > 0.05; males: *t* = 0.394–1.917; d.f. 13; P > 0.05). Therefore, the EAG responses of each sex to the same stimulus were merged irrespective of the collection site.

**Figure 1:**
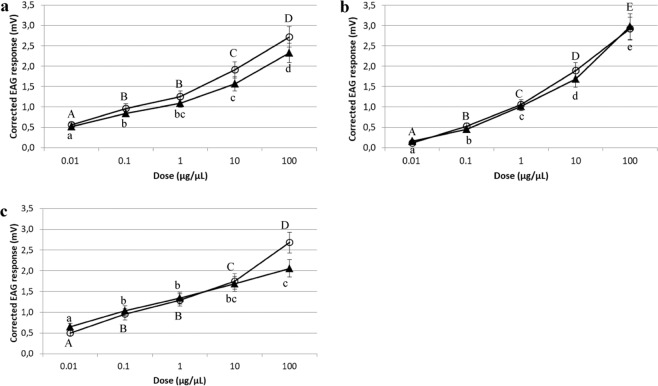
EAG dose-response curves of *Philaenus spumarius* to ascending doses of the three EOs. Adult males (▲) and females (○). A = *Pelargonium graveolens* EO; B = *Cymbopogon nardus* EO; C = *Lavandula officinalis* EO. Vertical bars represent S.E. Within each sex, EAG responses to different doses of each EO, were compared using a non-parametric Kruskal–Wallis test for multiple independent comparisons, with subsequent pair-wise Mann–Whitney U-test comparisons. Capital letters refer to females; lower case letters refer to males. Different letters indicate significant differences (P < 0.05).

All EOs tested elicited EAG dose-dependent responses. For both sexes, the activation threshold was 0.01 µg/µL for *P*. *graveolens* and *L*. *officinalis* EOs and 0.1 µg/µL for *C*. *nardus* EO (P < 0.05; one-sample *t*-test). For all EOs tested, female and male EAG responses increased from 10 to 100 µg/µL indicating no saturation of antennal receptors at the lower dose. Kruskal-Wallis test followed by pairwise Mann-Whitney U-test comparisons revealed significant differences in the EAG responses to different concentrations of each EO both in females (*P*. *graveolens* H = 43.340; df 4; P < 0.001; *C*. *nardus* H = 61.070; df 4; P < 0.001 and *L*. *officinalis* H = 49.607; df 4; P < 0.001) and males (*P*. *graveolens* H = 44.570; df 4; P < 0.001; *C*. *nardus* H = 56.792; df 4; P < 0.001 and *L*. *officinalis* H = 33.332; df 4; P < 0.001) (Fig. 1).

There were no significant differences between male and female mean EAG responses to each dose of *P*. *graveolens* (*t* = 0.347–1.262; d.f. 27; P > 0.05), *C*. *nardus* (*t* = 0.119–0.895; d.f. 27; P > 0.05) and *L*. *officinalis* (*t* = 0.211–1.944; d.f. 28; P > 0.05) with exception for the 100 µg/µL of this latter EO that elicited female EAG responses that were significantly higher than those of males (*t* = 2.452; d.f. 28; P < 0.05) (Fig. 1).

**Figure 2:**
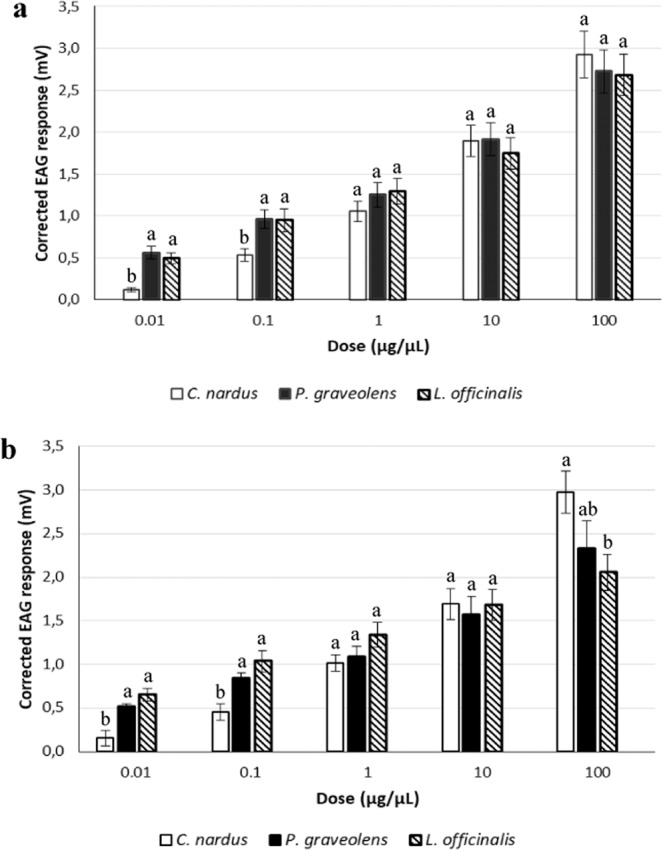
EAG response of *Philaenus spumarius* to the same doses of the three EOs. Adult females (**a**) and males (**b**). For each dose tested, the EAG responses of each sex to the three EOs were analysed by a non-parametric Kruskal–Wallis test for multiple independent comparisons, with subsequent pair-wise Mann–Whitney U-test comparisons (P < 0.05). Different letters indicate significant differences (P < 0.05).

Kruskal-Wallis test followed by pairwise Mann–Whitney U-test comparisons (P < 0.05) revealed significant differences among the EAG responses to the 0.01 and 0.1 µg/µL doses of the three EOs both in males (H = 22.453–15.979; df 2; P < 0.001) and females (H = 22.332–7.900; df 2; P < 0.001) (Fig. 2).

### EOs olfactometer bioassay

**Figure 3:**
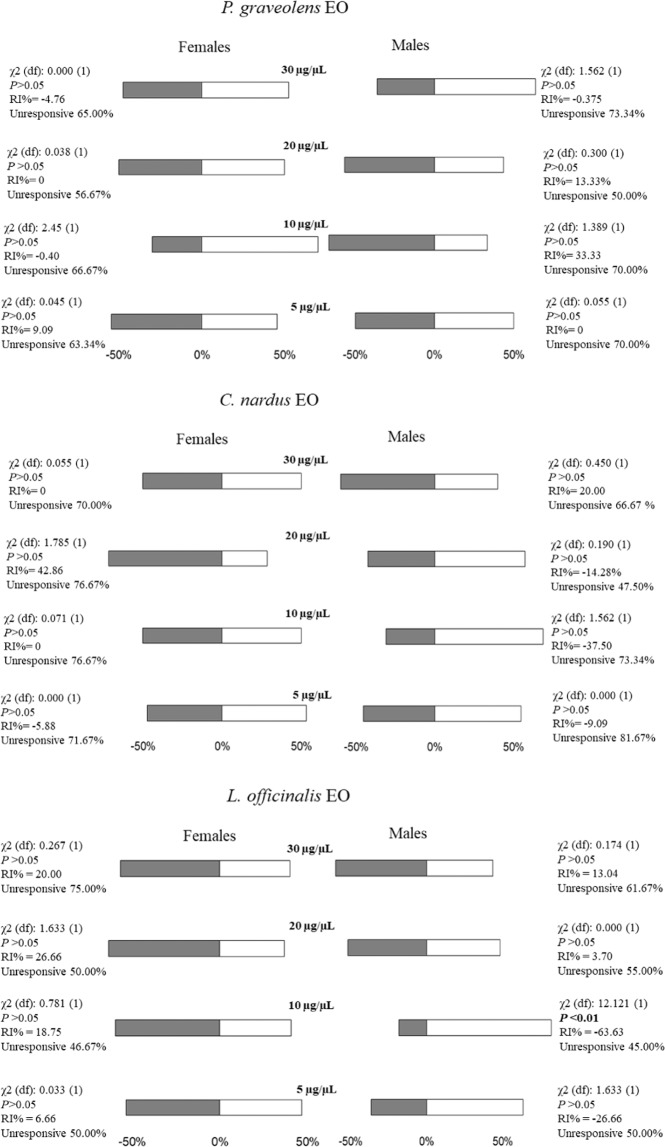
Y-tube behavioural response of *Philaenus spumarius* to volatile compounds emitted by the EOs: *Pelargonium graveolens*, *Cymbopogon nardus*, *Lavandula officinalis*, tested at four doses. Grey bars represent the percentage of females or males who chose the arm with the mineral oil (control), among insects who carried out the first choice. White bars represent the percentage of females or males who chose the arm with the stimulus in evaluation (essential oil), among insects who carried out the first choice. The insects that failed to make a choice within the first 15 min were utilised to calculate the unresponsive percentages. The repellency index was calculated as RI = (C − T/C + T) × 100, where: C = the number of *P*. *spumarius* in control arm; T = the number of *P*. *spumarius* in stimulus arm. RI varying from −100 (total attractiveness) to +100 (total repellency), with 0 meaning no effect. The Chi-square test with the Yate’s continuity correction for small sample sizes was used to determine significant difference between the observed and expected frequency of the insects choosing the stimulus (essential oils) and the control (mineral oil).

In Y-tube olfactometer, *P*. *spumarius* adults moved frantically essentially by walking. More than 45% of males and females were unresponsive towards all concentrations of EOs tested (Fig. 3). The number of males or females choosing the treatment arm were not significantly different from that of males or females in the control arm for any concentration of the three EOs tested, except for the 10 µg/µL concentration of *L*. *officinalis* EO that induced a significantly higher (χ^2^ = 12.121, P < 0.01, df = 1) number of males in the treatment arm compared to the control one. In both sexes, no significant differences were found in the time spent in each arm of the olfactometer towards any concentration of EOs tested.

### Plants olfactometer bioassay

**Figure 4:**
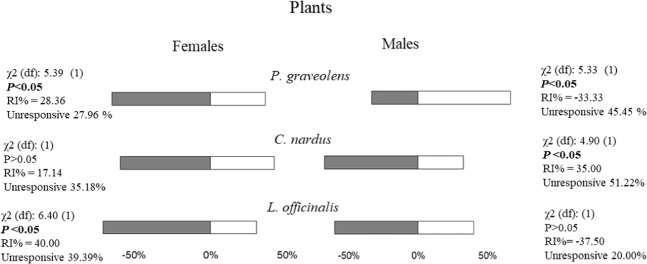
Y-tube behavioural response of *Philaenus spumarius* adults towards the selected plants. Grey bars represent the percentage of females or males who chose the arm with purified air (control), among insects who carried out the first choice. White bars represent the percentage of females or males who chose the arm with the stimulus in evaluation (plants of *Pelargonium graveolens*, *Cymbopogon nardus* and *Lavandula officinalis*), among insects who carried out the first choice. The repellency index was calculated as RI = (C − T/C + T) × 100, where: C = the number of *P*. *spumarius* in control arm; T = the number of *P*. *spumarius* in stimulus arm. RI varying from −100 (total attractiveness) to +100 (total repellency), with 0 meaning no effect. The insects that failed to make a choice within the first 15 min were utilised to calculate the unresponsive percentages. Chi-square test was used to determine significant difference between the observed and expected frequency of the insects choosing the stimulus and the control.

Across all experiments, about 60% insect (both sexes), made a choice within the maximum time allowed (15 min). The only differences between the overall responses of males and females (Fig. 4) were for *P*. *graveolens*, resulted attractive for males (χ^2^ = 5.33, P = 0.021, df = 1), and repellent for females (χ^2^ = 5.39, P = 0.020, df = 1). A general repellence was also recorded towards *L*. *officinalis* and *C*. *nardus* (both sexes), even though it was significant only for females towards lavender (χ^2^ = 6.4, P = 0.011, df = 1) and for males towards citronella (χ^2^ = 4.9, P = 0.027, df = 1) (Fig. 4).

### Plant wind tunnel bioassay

**Figure 5:**
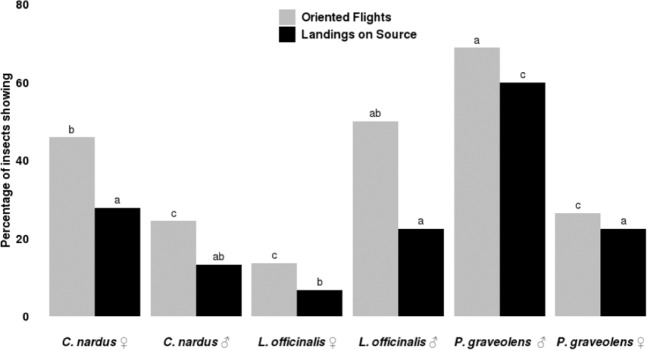
Wind tunnel behavioural response of *Philaenus spumarius* adults towards *Cymbopogon nardus*, *Lavandula officinalis* and *Pelargonium graveolens* plants. Data collected were analysed by using G-test of independence. Different letters indicate significant differences between plants tested within each behaviour observed. For each plant, 60 females and 60 males were tested.

Across all experiments, 87% of *P*. *spumarius* (both sexes) made a choice within 10 min. Overall 39% of the adults flew upwind over 50 cm towards target plants and landed (26%) on them. Wind tunnel results were in line with those recorded in olfactometer (compare Figs. 4 and 5). In detail, males resulted more attracted than females by *P*. *graveolens* (69% *vs* 26% oriented flights) (G test, χ^2^ = 17.433, df = 1, P < 0.01). Similarly, significant differences between male and female responses were recorded for *C*. *nardus* (24% *vs* 46% oriented flights; G test, χ^2^ = 4.202, df = 1, P = 0.040) and *L*. *officinalis* (50% *vs* 14% oriented flights; G test, χ^2^ = 13.442, df = 1, P < 0.01) (Fig. 5).

## Discussion

The sustainability of modern agriculture largely depends on the approaches followed for plant protection. Natural-derived compounds are increasingly replacing synthetic pesticides to satisfy the requests of consumers asking for cleaner products^28,34,35^. In this scenario, volatile organic compounds regulating the foraging behaviour of pests and antagonists are receiving increasing attention^36,37^. In this paper we explored the behavioural responses of the main vector of *X*. *fastidiosa*, the spittlebug *P*. *spumarius*, to a selection of essential oils (EOs) and aromatic plants. The volatile compounds associated to them have been long since tested particularly for the sustainable management of Diptera and Coleoptera^38,39^.

In the present study, the selected EOs elicited antennal responses in both males and females of *P*. *spumarius* demonstrating the capability of the peripheral olfactory system of both sexes to perceive volatile compounds present in these EOs. Male and female antennae also showed to be sensitive to variations in concentrations of EOs, as shown by the dose dependent EAG responses elicited by increasing concentration of each EO. This result supports the idea that EO volatiles may act as long-distance cues to *P*. *spumarius* adults. Moreover, differences in the male and female perception of some concentrations of the three EOs demonstrated the capability of the insects’ olfactory system to perceive them selectively. All these observations further confirm the antennal responsiveness of adult *P*. *spumarius* to a wide range of VOCs^33^. Moreover, in this study, spittlebug adults collected from different herbaceous plants in two different areas gave similar EAG responses to different concentrations of the three EOs thus suggesting a common innate responsiveness to the stimuli tested.

These results have important consequences for the use of EOs for plant protection from insects that act as vectors of phytopathogens independently from the sex. Assessment of dose response curves relative to the EOs tested with indication of the activation threshold and the saturation level of perception represents a fundamental information for field application of these products or related plants within IPM protocols. In fact, last-generation dispensers guarantee that these compounds are released at specific rates that assure a specific level of concentration all along the day^18,40^.

However, antennal recognition by itself does not indicate which is the final response in terms of behaviour, being it either attraction to (usually a host plant) or repellence from (usually a non-host plant). For this reason, we planned behavioural bioassay to assess the elicited behaviour at close (olfactometer) and long range (wind tunnel) towards the same EOs and related plants. These bioassays clearly indicate that male and females of *P*. *spumarius* respond differently to the same volatile blend coming from either EO or entire plant, probably as a result of the different role played by the same stimuli in the ecology of males and females. The high percentage of non-response to EOs in olfactometer could have been due to the concentration of volatile compounds within the device even though standard protocols have been used. Another explanation could be the absence of “companion” compounds in the blend released by the whole plant that play a role in the behaviour of the spittlebug^41^. There is some evidence that perception of blends of plant volatiles plays a pivotal role in host recognition, non-host avoidance and ensuing behavioural responses as different responses can occur to a whole blend compared to individual components. Components of host (and non-host) plant blend may not be “recognised” when perceived outside the context of that blend^41,42^. Among the responding insects to each EO a general frenzy was noted with several jumping and flight attempts which were not noted testing the real plants. Nonetheless, even in EO olfactometer bioassay, a clear attraction was recorded for males towards lavender. This result was confirmed by testing lavender plants at close (olfactometer) and long (wind tunnel) range. Indeed, these plants resulted clearly repellent for females and attractive for males whilst the reverse occurred for geranium. The EOs of these two plant species displayed a similar repellent activity against *Empoasca vitis* adults, a main pest of tea in China, even though no sexual differences were noted^43^. To our knowledge, this is the closest result to ours in terms of insect species (a leafhopper) and semiochemicals tested (EO). Conversely, whilst no response to citronella was recorded for *E*. *vitis*, this plant proved repellent for males of *P*. *spumarius* at close (olfactometer) and long range (wind tunnel) and attractive towards females but only at long range (Figs. 3–5). Both lavender and geranium EOs have been reported to alter the behaviour of other pest insects including pollen beetles^38^ and midges^39^. These results, in agreement with ours, indicate that lavender can be truly considered as a possible candidate in different plant-pest systems to reduce (in time and space) pest populations on main crops and in turn the use of synthetic insecticides. The concentration of target pests in restricted areas of the field represents a desirable strategy to improve the efficacy of any insecticidal treatment, reducing both the time and the quantity of toxic compounds needed to attain a satisfactory control. However, considering that both sexes of *P*. *spumarius* can act as vectors of *X*. *fastidiosa*, and that plants or EOs here tested elicited a differential response among sexes, a combination of companion plants (or EOs) should be planned to build up either a push-and-pull or attract-and-kill strategy in olive orchards. For example, citronella together with either lavender or geranium, placed along olive orchard borders, could attract and concentrate large number of female and male spittlebugs in a zone where they can be more easily treated.

The behavioral effects of EOs and plants, here tested, required to be confirmed to adult *P*. *spumarius* in a crop protection situation where interactions among volatiles from different plants almost certainly occur. Moreover it remains to be assessed whether the EOs here tested, directly applied on olive trees retain their efficacy as repellents and whether they have a phytotoxic effect. In fact, EOs have been generally used as natural insecticides than as modifiers of the foraging behaviour of key pests in horticultural systems. For example, the EOs of thyme have dramatic impact on the survival of all instars of *Bemisia tabaci*, particularly its eggs, whose mortality reached 74.5%^44^. Testing the direct, toxic effect of *P*. *graveolens*, *C*. *nardus* and *L*. *officinalis* EOs on eggs, preimaginal instars and adults of *P*. *spumarius* could enlarge their possible use in the sustainable control of *X*. *fastidiosa* vector. Recently, insecticidal effect of sweet orange EOs has been noted in field trials^31^.

The control of generalist species such as *P*. *spumarius* must rely on an integrated approach. In this context, the contribution of EOs or companion plants can be significant only if associated to the use of resistant varieties and to the application of agronomic measures such as the periodic mowing of herbaceous plants under canopy^45^. It is also worth noting that the possible reduction of land surface dedicated to olive trees, for planting companion plants at the edge of the field, can be largely compensated by the extra income deriving by harvesting these aromatic plants, highly requested by cosmetic and medical industries. This strategy is becoming increasingly followed in vineyards all over Europe to control key pests^46^.

*X*. *fastidiosa* has proved to have a dramatic impact on Mediterranean olive cultivation and sustainable methods to control its vectors are highly needed to contrast its rapid spread. This study evaluated the response of adult middle spittlebugs to chemical cues in the absence of visual stimuli and on the whole showed a good correlation between the bioactivity of odour sources and the negative or positive insect host preferences previously observed. Highlighting the importance of chemical signals in the host-plant location by this insect. Next steps in the identification of EOs or aromatic plants that can contribute to the reduction of vector populations include widening the number of species tested and starting field applications. The latter will benefit in first instance of the results presented here.

## Materials and Methods

### Insects

Adults of *Philaenus spumarius* were collected by sweeping net weekly from natural populations during September-November 2018 in Molise and Campania (Italy). In detail, in Molise they were collected on *Medicago sativa* in Tufara (Campobasso, Molise, Italy 41°28′43.2″N, 14°54′40.2″E). In Campania, adults were collected in a wild grass pasture in Tocco Caudio (Benevento, Campania, Italy, 41°06′12.5″N 14°38′05.9″E). Soon after collection, insects were transferred onto *Vicia faba* plants in aerated cages (Vermandel^®^, 100 × 70 × 70 cm) kept in laboratory, at natural temperature and photoperiod. Host plants were periodically refreshed. Insect were sexed before running any experimental bioassay.

### Essential oils and plants

EOs and plants to be tested were selected on the basis of the results of previous studies mainly focused on their repellence to mosquitoes^47–52^ and leafhoppers^43^. Essential oils of *Pelargonium graveolens* L’Herit flowers, *Cymbopogon nardus* Rendl. grass and *Lavandula officinalis* Chaix flowers were purchased from Solimè s.r.l. (Cavriago, Reggio Emilia, Italy).

Plants of *P*. *graveolens*, *C*. *nardus* and *L*. *officinalis* were purchased at the garden centre Mignogna (Toro, Campobasso, Italy) and were all about 2-year old.

### Electroantennographic recording (EAG)

The EAG responses of *P*. *spumarius* male and female antennae to increasing concentrations (0.01–100 µg/µL) of the three EOs were measured by the EAG technique similar to that used in previous studies^33,53^. In order to prevent the rapid evaporation of test compounds, EOs were dissolved in mineral oil (Sigma-Aldrich, Milan, Italy) and stored at −20 °C until needed.

For the test, a single adult was dissected between the head and the thorax and a glass micropipette (0.2–0.3 mm i.d.) filled with 0.1 M KCl solution, acting as the indifferent electrode, was inserted into the head. The last antennal segment was put in contact with the end of a similar pipette which provided the recording electrode. AgCl coated silver wires were used to maintain the electrical continuity between the antennal preparation and an AC/DC UN-6 amplifier in DC mode connected to a PC equipped with the EAG 2.0 program (Syntech). A stream of charcoal-filtered humidified air (500 ml/min) was directed constantly onto the antenna through a stainless steel delivery tube (1 cm i.d.) with the outlet positioned at approximately 1 cm from the antenna. Twenty five microliters of each stimulus were absorbed onto a filter paper (Whatman No. 1) strip (1 cm × 2 cm) inserted in a Pasteur pipette (15 cm long) and used as an odour cartridge. Over 1 s, 2.5 cm^3^ of vapour from an odour cartridge were blown by a disposable syringe into the air stream flowing over the antennal preparation. Intervals between stimuli were 1 min. Standard (25 μl of (*Z*)-3 Hexen 1 ol) and control (25 μl mineral oil) stimulus were applied at the beginning and at the end of the experiment. In addition, the standard stimulus was applied after each group of two test odours, to evaluate the gradual decrease in the antennal sensitivity over time.

For each population (Campobasso, Benevento), the EAG responses were recorded from at least 13 antennae of different males and females.

The amplitude (mV) of the EAG response to each test stimulus was adjusted to compensate for solvent and/or mechanosensory artefacts according to Raguso & Light^54^. To compensate for the decrease of the antennal responsiveness during the experiment, the resulting EAG amplitude was corrected according to the reduction of the EAG response to the standard stimulus^55^. The Student’s *t*-test for independent samples were used to compare the mean EAG responses of specimens of each sex collected in the two different areas (Campobasso, Benevento) and those of males and females of the same area.

In dose–response curves, the activation threshold was considered to be the first dose at which the mean response was higher than “0” value using Shapiro-Wilk test for normality followed by one-sample Student’s *t*-test (P = 0.05)^33^. Saturation level was taken as the lowest dose at which the mean response was equal to or less than the previous one^56^. The non-parametric Kruskal-Wallis test for multiple independent comparisons followed by pairwise Mann–Whitney U-test comparisons (P < 0.05) were used to compare the mean EAG responses of each sex to different doses of each EO and to the same dose of different EOs.

Data were processed by Statistical Package for Social Sciences (SPSS), version 25.0 per Windows software (SPSS Inc., Chicago, IL).

### Olfactometer bioassay with EOs

A glass Y-tube olfactometer (each arm 23 cm long at 75° angle, stem 30 cm long, 3.0 cm i.d.), similar to that described in Germinara *et al*.^57^, was used to examine the behavioural responses of *P*. *spumarius* to EOs. Each arm of the Y-tube was connected to a glass cylinder (9 cm long, 3.0 cm i.d.) as an odour source container. The apparatus was put into an observation chamber (90 × 75 × 40 cm) and illuminated from above by two 36-W cool white fluorescent lamps providing uniform lighting (2500 lux) as measured by a photo-radiometer (HD 9221 Delta OHM). A purified (activated charcoal) and humidified (bubble bottle) airflow set at 12 cm^3^/min by a flowmeter (Alltech Digital Flow ChecKTM), was pumped through each arm. Bioassays were run between 9.00 a.m. and 18.00 p.m. and lasted 15 minutes each. In the centre of one glass cylinder a filter paper disc (1 cm^2^) soaked with 5 μl of the testing solution was suspended while in the other cylinder a similar disc loaded with 5 μl of mineral oil (control) was placed. One insect was released into the stem of the apparatus and its preference for one of the two odour sources was recorded. A choice was recorded when the insect walked up the first 3 cm of an arm of the olfactometer and remained beyond that line for at least 10 sec. Insects that failed to make a choice within 15 min were considered unresponsive. Odour sources were presented in a random order and the olfactometer was rotated 180° after every day, to correct for any unforeseen asymmetrical bias in the setup. Each EO was tested at 5, 10, 20, 30 µg/µL and for each concentration tested, 60 females and 60 males were tested during 7 different days.

For each test stimulus, a repellency index was calculated as RI = (C − T/C + T) × 100, where: C is the number of insects responding to the control and T is the number responding to the stimulus. RI can vary from −100 (total attractiveness) to +100 (total repellency)^58^. The Chi-square test with the Yate’s continuity correction for small sample sizes was used to determine significant difference between the observed and expected frequency of the insects choosing the stimulus and the control^59^. Wilcoxon paired signed ranks test was used to analyse the differences between the time spent by insects in the two arms.

Statistical analyses were performed using SPSS 25.0 per Windows software (SPSS Inc., Chicago, IL, USA).

### Olfactometer bioassay with plants

A dynamic airflow Y-tube olfactometer (stem length 9 cm, arm length 8 cm, diameter 1 cm) as described in detail by Cascone *et al*.^60^ was used to test the behavioural responses of *P*. *spumarius* adults towards the selected plants. A stream of purified air was split into two equal air streams. Each airflow was humidified by passing through a jar with distilled water and connected to a glass container (height 45 cm, diameter 20 cm) holding an odour source (a potted plant) through an inlet situated in the lid. The containers were sealed with a Viton O-ring and a metal clamp. Before placing the plants in the glass container, the pots were covered with an aluminium foil. Air from each odour container was subsequently led into one of the arms of a glass Y-tube olfactometer and set at 100 ml min^−1^ by flowmeters. A choice was considered made when the insect reached either the final 2-cm part of an arm of the Y-tube or the trapping bulb connected to the final part of each Y-tube arm. One insect was released into the apparatus and its preference for stimulus (odours of potted plant) or control (purified air) was recorded after 15 min. For each odour source tested, at least 60 females and 60 males were used over 7 different days. Odour sources were presented in a random order in the glass containers and the olfactometer was rotated 180° after every day, to correct for any unforeseen asymmetrical bias in the setup. For each odour test, a repellency index was calculated as RI = (C − T/C + T) × 100, where: C is the number of insects responding to the control and T is the number responding to the stimulus. RI can vary from −100 (total attractiveness) to +100 (total repellency)^58^. Chi-square test was used to determine significant difference between the observed and expected frequency of the insects choosing the stimulus and the control. Analysis was conducted using the R statistics programming environment 3.3.3 (R Development Core Team, 2012).

### Wind tunnel bioassay with plants

Wind tunnel bioassay was run as described in detail by Guerrieri *et al*.^61^ but the insect releasing vial was replaced by a wood tongue depressor allowing a better grip for adult *P*. *spumarius*. Each adult was observed for a maximum of 10 minutes. The same plants used for olfactometer biossay were assessed in wind tunnel. For each selected plant species, 60 females and 60 males were individually tested over 7 different days and the percentage of insect exhibiting oriented flight and landing on the target was calculated. Insect behaviour was recorded as “Oriented flight” when the insect flew towards the target landing either on it or within 5 cm away from it. Similarly, it was recorded as “Landing on target” when females landed on plant. The number of insects responding, as oriented and non-oriented flight to and landing on each target was compared by a G test for independence, as described in Sokal and Rohlf^62^, using the pairwise G test procedure (package RVAideMemoire, 2017) and R statistics programming environment 3.3.3 (R Development Core Team, 2012).

### Ethical approval

This article does not contain any studies with human participants or animals (vertebrates) performed by any of the Authors.

## Supplementary information

Dataset 1.
